# An Analysis of the School Catering Management System of Thailand Through HiAP's Four Pillars

**DOI:** 10.1002/hpja.70114

**Published:** 2025-11-20

**Authors:** Nanoot Mathurapote, Tipicha Posayanonda, Khanitta Sae‐iew

**Affiliations:** ^1^ The National Health Commission Office (NHCO) Nonthaburi Thailand

**Keywords:** health in all policies, health system, HiAP, participation, school catering, Thailand

## Abstract

**Issue Addressed:**

Despite the 1992 Primary School Lunch Program Fund Act, Thai children were nutritionally deficient and had low fiber consumption. Although having three ministries—public health, education and interior—involved with the school catering management system, the coordination was inadequate. In 2014, Thailand National Health Assembly (NHA) discussed this issue for better multi‐sectoral solutions, and it was considered successful. This study explored the application of WHO Health in All Policies (HiAP)'s four pillars in the implementation of the NHA resolutions on ‘School Catering Management System’. Enabling factors and challenges were also studied.

**Methods:**

Literature review, focus group discussions and in‐depth interviews of stakeholders at national and provincial levels.

**Results:**

Analysis of the application of the HiAP approach on the NHA resolution on the school catering management system showed that it was complying with the four pillars: (1) governance and accountability; (2) leadership at all levels; (3) ways of working; and (4) resources, financing and capabilities. The NHA, Cabinet resolutions, and related official and unofficial mechanisms, as well as leadership were considered enabling factors leading to strong collaboration on school catering management.

**Conclusions:**

To apply a health lens in non‐health agencies using HiAP, it necessitates working through all of HiAP's four pillars. The legitimacy of governance bodies, ways of working and leadership along the process are enabling factors to improve coordination of this case with multi‐sectoral collaboration. However, there are still challenges.

**So What?:**

HiAP's four pillars are useful for the policy process. It needs to analyse what pillar is weak and needs to improve for its sustainability.

## 1 Introduction

School catering is critical for child development and well‐being, as it shapes health habits during formative years. Despite the 1992 Primary School Lunch Program Fund Act, Thai children were nutritionally deficient and had low fibre consumption [1]. Although the school catering management system in Thailand emphasised food quality, safety and nutrition education, with involvement from the National Food Committee, the Ministries of Public Health (MoPH), Interior and Education, collaboration issues among these bodies were limited and could hinder the progress.

For better solutions, in 2014 the issue of school catering management system was proposed to Thailand National Health Assembly (NHA) [2, 3, 4, 5], which started in 2008 according to the National Health Act 2007 as a multi‐sectoral and stakeholder platform for fostering collaboration between health and non‐health sectors to address complex health‐related issues.

The school catering management system was adopted as an NHA resolution [6] by members of NHA from government agencies, civil societies and academia, focusing on: (1) policy—elevating school catering to the national agenda; (2) quality assurance and standards—creating mechanisms for managing and monitoring and (3) information systems—developing databases to support management.

Following the adoption, the NHA Organizing Committee submitted the NHA resolution to the National Health Commission (NHC), which presented it to the Cabinet. In 2015, the Cabinet issued a resolution supporting the NHA's recommendation for multi‐sectoral collaboration in managing the school catering management system [7]. The NHA Resolution Follow‐Up Committee started driving the resolution into action by seeking collaboration with multi‐actors.

After the Cabinet resolution, the National Food Committee (NFC) established a sub‐committee dedicated to managing food and nutrition in schools. This marked the first time the NFC had formed a sub‐committee specifically focused on food management within a school setting. The sub‐committee's role aligned with the objectives of the NHA resolution, further cementing the issue of school catering as a national priority. In addition, the sub‐committee's scope expanded to include communities and other educational institutions like child care centres, reflecting a broader commitment to food safety and nutrition beyond schools.

In 2018, the Cabinet reaffirmed the NHA's stance, directing Surin Province, which was renowned for organic farming practices, to implement the school catering management system resolution throughout the province [8]. This extended the issue to include the Ministry of Agriculture and Cooperatives to promote organic and safe farming practices, and to link school catering with local agricultural and economic development.

In 2022, the Cabinet approved an increase in lunch subsidy for students from kindergarten through Grade 6, as proposed by the Ministry of Education (MoE). This adjustment aimed to ensure that school lunch programmes could meet the demands and enhance the nutritional quality of meals provided to students [9].

At the operational level, ‘Manual on School Catering Management System for Nutritious and Safety Food’ [10] and ‘Online Thai School Lunch Program’ were developed to provide guidelines and budget‐friendly menu options to ensure having school meals with safety and nutrition standards. Additionally, ‘Funding Management Measures for Providing Lunch at Primary Schools’, were introduced in 2020 as a framework designed to streamline the budgeting process for school lunches to ensure that the budgeting process would consistently support the provision of quality and safe food for students.

The implementation of this NHA resolution on school catering management system is considered successful as it has achieved key outputs stated in the resolution [11, 12]. This study explored the application of the World Health Organization (WHO)'s Health in All Policies (HiAP) approach on the resolution using HiAP's four pillars: (1) governance and accountability, (2) leadership at all levels, (3) ways of working and (4) resources, financing and capabilities [13] as a framework of analysis. Enabling factors and challenges to ensure implementation of the resolution from the national to provincial levels were also studied.

## 2 Methodology

This study utilised a combination of literature review, two focus group discussions at the national and provincial levels, a total of 21 participants, and in‐depth interviews of 11 interviewees. The participants and interviewees, from government officials, researchers, civil societies and communities, were selected based on their roles in the school catering management policy process. Content from the discussions and interviews was collected and its consistency with WHO HiAP's four pillars was analysed. Enabling factors and challenges to ensure implementation of the resolution were also studied.

## 3 Results and Discussion

### 3.1 Mechanisms at the National and Provincial Level

#### 3.1.1 National Level

Many mechanisms played vital roles in ensuring the implementation of the school catering management system resolution: The NHA Organizing Committee allowed related stakeholders from various sectors to understand the issue, discuss and adopt the resolution; the NHA Resolution Follow‐Up Committee monitored and ensured multi‐sectoral implementation of the resolutions; the National Health Commission (NHC), chaired by the Prime Minister, secured political commitment and implementing the resolution; and the National Food Committee realised the importance of the issue and established a specific sub‐committee responsible for the resolution.

**FIGURE 1:**
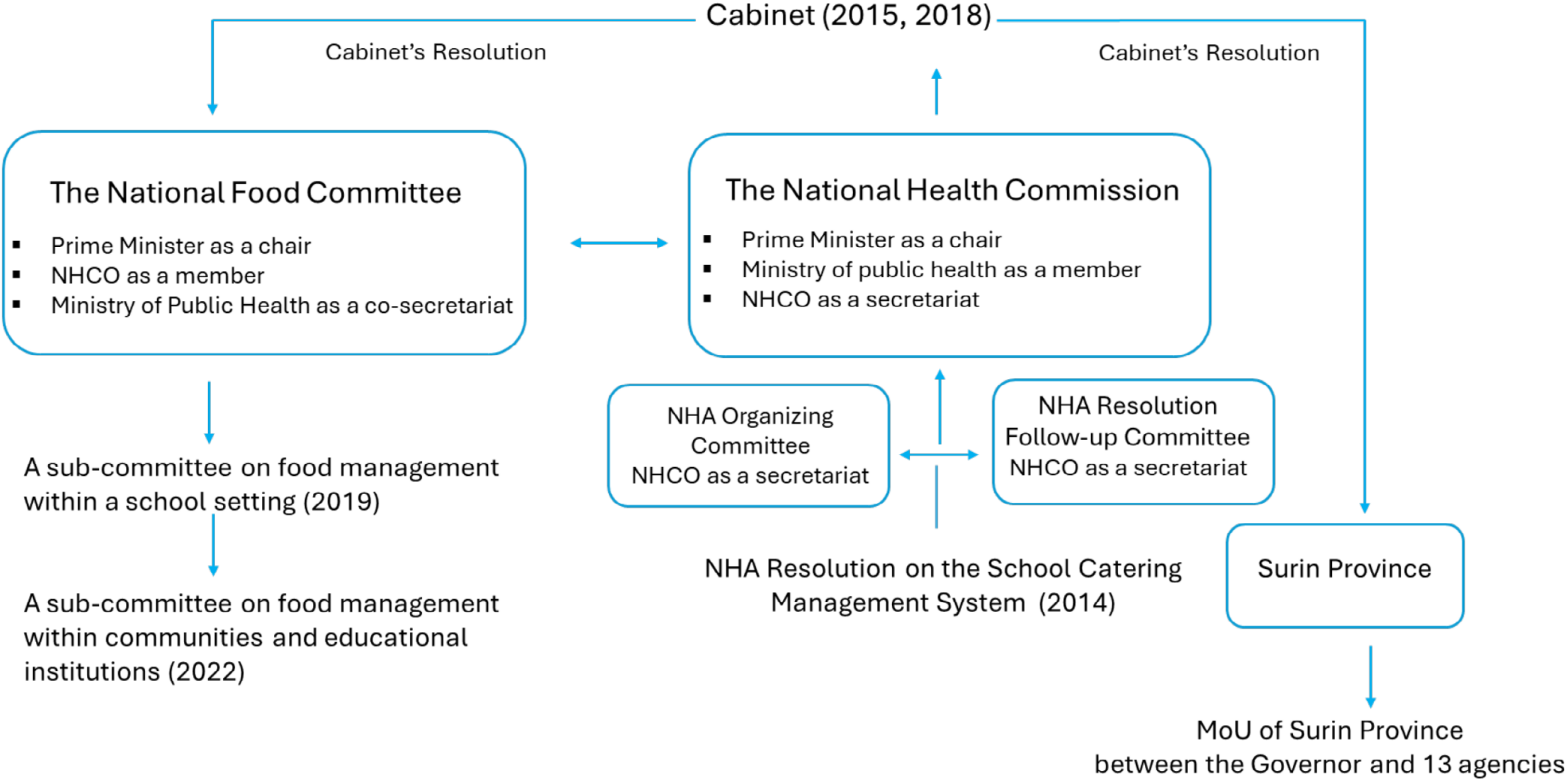
Health in all policies on school catering system management at the policy level.

It was found that the National Health Commission Office (NHCO), which served as secretariat for the three above mentioned national health mechanisms, was also a member of the National Food Committee. This connection between governance mechanisms as cited in the Figure 1 likely facilitated better coordination in information flow, policy implementation and promoting healthy nutrition across educational settings.

#### 3.1.2 Provincial Level

According to the Cabinet resolution specifically directed to Surin Province on the school catering management system, Surin Province employed a multi‐sectoral approach guided by the Memorandum of Understanding (MoU) between Provincial Governor and 13 agencies including local authorities and civil society organisations (CSOs) in the province. An informal working group led by CSOs related to organic farming was formed to work with such official mechanisms as Surin District Health Board and Surin Provincial Health Assembly Committee showing multi‐sectoral collaboration and a combination of formal and informal mechanisms at the provincial level.

It was highlighted that expertise and long‐standing efforts of Surin's CSOs in organic farming was a factor leading towards provincial government's trust and a successful implementation of the school catering management system in the province.

### 3.2 Governance and Accountability

It was found that multi‐sectoral mechanisms, that is, NHA Organizing Committee, National Health Commission, which were put in place by the National Health Act 2007, and other related national mechanisms such as NHA Resolution Follow‐Up Committee, National Food Committee, Sub‐Committee on food management, were supportive government structures leading to policy and commitment as well as accountability, as shared vision and objectives were clearly set in the NHA resolution.

Further, the integration of the National Health Commission and the National Food Committee, with involvement from the National Health Commission Office and the Ministry of Public Health, ensured smooth information flow and joint planning, aiding both the drafting, implementing and monitoring of the resolution.

In addition, the Cabinet resolutions in 2015 and 2018 which aligned with the NHA resolution legitimised the school catering management system policy, whereas the MoU in Surin Province enabled multi‐sectoral collaboration and implementation throughout the province. It was shown that both the Cabinet resolutions and the MoU facilitated strong collaboration across operational level. Therefore, vertical and horizontal governance structures are both crucial for commitment and implementation of the school catering management system.

From the focus group discussions, most interviewees informed that the policy and legitimacy through the exist mechanisms in the governance structures were a key factor for the implementation. However, the academia suggested that in order to reach an effective monitoring, having specific indicators to measure outputs and outcomes of the school catering management system should be put in place.

### 3.3 Leadership at All Levels

Governance is not enough, ensuring HiAP requires leadership at all levels. At national level, MoPH was the leader pushing the school catering management system to the NHA. After the Cabinet resolution Department of Health of MoPH (under the Sub‐committee on food management) started a discussion with Ministry of Education (MoE) and academia from Mahidol University and National Electronics and Computer Technology Center (NECTEC) to develop ‘Manual on School Catering Management System for Nutrition and Safety Food’ and ‘Online Thai School Lunch Programme’. Additionally, MoE proposed to the Cabinet the approval of an increase in lunch subsidy for students from kindergarten through Grade 6, whereas MoE together with Ministry of Interior (MoI) developed ‘Funding Management Measures for Providing Lunch at Primary Schools’. The manual, online programme and funding management measures were later recommended to be used in all schools over the country. At present, although not all schools are using the manual and the online programme, many of them are. It depends on the head of each school paying attention on these guidelines.

For schools in Surin province, both the manual and online programme were used. In addition, this study showed that success of the implementation of the NHA resolution had been largely driven by the leadership of both provincial government, public health personnel and civil society organisations, particularly the Provincial Health Assembly Network which played an important role as a collaboration point that could bring together people across sectors within the province to continuously drive the policy into action. While similar networks exist in most provinces, not all possess the expertise or interest in advancing this agenda. Therefore, success depended on local stakeholders adopting the initiative as their own, demonstrating the importance of leadership and long‐term commitment, which were not achieved solely through Cabinet resolutions or regulations.

An interviewee from CSO in Surin province emphasised that *‘leadership was built from attitude and understanding on the importance of the issue’*. Because of long standing commitment on organic farming and food safety in Surin, implementation of the school catering system can easily expand a provincial wide.

### 3.4 Ways of Working

The combination of formal and informal mechanisms fostered relationships, collaboration and continuity across sectors. The participatory NHA process brought together ministries, technical people and CSOs, allowing fruitful dialogue, promoting relationships and shaping direction and goals. The NHA Resolution Follow‐Up Committee played a crucial role in maintaining multi‐sectoral cooperation and furthering good relationships and HiAP implementation. In Surin, long‐standing collaboration among the governor, local governments, public health personnel and CSOs—rooted in organic farming initiatives—built trust and facilitated a co‐design approach towards shared goals and led to a smooth transition towards implementing a healthy school catering system in the province.

There was an issue on information sharing and knowledge exchange raised by interviewees from the public health sector that in the ways of working through multi‐sectoral mechanisms at any level, evidence and data were necessary to be informed in order to plan, decide, and put in place suitable activities for achieving the agreed goals.

### 3.5 Resources, Financing and Capabilities

The Manual on School Catering Management System for Nutritious and Safety Food and the Funding Management Measures provided cross‐sectoral collaboration support. The Online Thai School Lunch Programme offered a practical solution for schools with limited resources to provide quality lunches.

Capacity building to school teachers, parents, cooks and relevant agencies, were needed, but should not limit to how to manage school lunch and funding or how to use online Thai school lunch programme, Attitude and understanding on food quality and safety should be attached in these capacity building programmes.

This study showed that at national level, the exist national collaborative mechanisms were fully supported by such government agencies as MoPH, MoE and NHCO on budget and responsible persons for the sustainable implementation of the school catering management. It was found that in Surin, the Provincial Health Assembly Committee demonstrated its capability to facilitate collaboration between CSOs and government agencies, showcasing how HiAP could be effectively supported by trusted and capable civil society actors.

From the study, HiAP's four pillars were analysed at the national and provincial levels as shown in Table 1.

**TABLE 1:** HiAP's four pillars and elements to drive the implementation of the school catering management system.

Pillars	Elements
Pillar 1: governance and accountability	National Health Commission NHA Organizing Committee NHA Resolution Follow‐Up Committee National Food Committee Sub‐committee on food management Surin Provincial Health Assembly Committee District Health Board NHA resolution in 2014 NHC resolution in 2015 Cabinet resolutions in 2015 and 2018 Memorandum of Understanding (MoU) in Surin Province
Pillar 2: leadership at all levels	Ministry of Public Health (MoPH) Ministry of Education (MoE) Mahidol University National Electronics and Computer Technology Center (NECTEC) Provincial government Provincial Health Assembly Network Civil society organisations in Surin province
Pillar 3: ways of working	National Health Assembly (NHA) with the National Health Commission Office (NHCO) as a secretariat functioned as a formal mechanism and organised in a participatory manner NHA Resolution Follow‐up Committee with NHCO as a secretariat maintained multi‐sectoral cooperation on the implementation of the issue Provincial Health Assembly functioned as an informal mechanism collaborating between government agencies and CSOs A combination of formal and informal mechanisms fostered relationship, collaboration and continuity across sectors Instead of laws and regulations, the MoU was used for multi‐sectoral commitment in the province
Pillar 4: resources, financing and capability	Manual on School Catering Management System for Nutrition and Safety Food developed by Ministry of Public Health and Ministry of Education Funding Management Measures for Providing Lunch at Primary Schools developed by Ministry of Education and Ministry of Interior Online Thai School Lunch Programme developed by Ministry of Public Health, Mahidol University and the National Electronics and Computer Technology Center (NECTEC) Budget and responsible persons for the sustainable work of related national collaborative mechanisms for the implementation of the school catering management supported by MoPH and NHCO Budget and responsible persons for the mechanisms at Surin province from government agencies and CSOs

### 3.6 Enabling Factors and Challenges

It was found that having the NHA and Cabinet resolutions with a clearly defined vision and objectives agreed upon by all stakeholders through collaborative approaches was legitimate and considered a key enabling factor that led to financial and personnel support and that was able to enhance incentives for the implementation. In addition, having the National Health Commission Office (NHCO) as a secretariat for the national health mechanisms: NHA Organizing Committee, National Health Commission and NHA Resolution Follow‐Up Committee, as well as having the NHCO as a member of the National Food Committee was another enabling factor to facilitate coordination in information flow and policy implementation.

Both the Cabinet resolutions and the MoU in Surin Province also enabled strong collaboration across operational level. Moreover, it was noted that long standing commitment on organic farming and food safety in Surin, could efficiently expand the implementation of the school catering management system in the province.

However, challenges were noted that specific indicators should be developed to measure outputs and outcomes of the school catering management system in order to reach an effective monitoring. Further, ways of working could be improved by increase in information sharing and knowledge exchange via exist mechanisms in order to plan, decide and develop suitable activities under any contexts that might be changed.

## 4 Conclusion

From the analysis of the application of the WHO's Health in All Policies (HiAP) approach on the NHA resolution on school catering management system resolution using HiAP's four pillars, it was found that the success of the implementation of the resolution complied with the four pillars as the legitimacy of governance bodies, for example, NHA mechanisms and resolutions endorsed by both the Cabinet and the NHC, as well as the provincial MoU, did elevate the issue to a national policy and draw multi‐sectoral collaboration. With the legitimacy of the established governance bodies, therefore, budget allocations, resources and capabilities certainly followed accordingly. The combination of formal and informal mechanisms, like the National and Provincial Health Assemblies and related mechanisms, was considered as ‘ways of working’ playing a key role in building trust among stakeholders and engaging various actors. Although leadership was found needed at both national and provincial levels with leaders having the attitude and mindset of collaboration together with an understanding of the importance of the issue, it is still challenging to continually build collective leadership for the sustainability of the implementation of the resolution at all levels in order to be expanded to other provinces and nationwide.

Therefore, to apply a health lens in non‐health agencies using HiAP approach, it necessitates to work through all HiAP's four pillars. From this study, further recommendations include development of specific output and outcome indicators for the school catering management system and improvement of information sharing and knowledge exchange through the exist collaborative mechanisms in order to respond to any changing current situation.

## Author Contributions

N.M., T.P. and K.S.‐i. developed the structure of this article and reviewed the literature. T.P. and K.S.‐i. conducted the interviews. N.M., T.P. and K.S.‐i. analysed the data and drafted the manuscript. N.M. and T.P. revised the drafted manuscript. All authors read and approved the final version.

## Disclosure

Disclaimer: Thailand Science Research and Innovation and National Health Commission Office does not have any influence on the design and findings of this study.

## Ethics Statement

This study was approved by the Institute for the Development of Human Research Protections, Thailand.

## Consent

The authors have nothing to report.

## Conflicts of Interest

The authors declare no conflicts of interest.

## Data Availability

The data that support the findings of this study are available on request from the corresponding author. The data are not publicly available due to privacy or ethical restrictions.
